# [*rac*-2-(1-Amino­eth­yl)phenyl-κ^2^ *C* ^1^,*N*](ethyl­endiamine-κ^2^ *N*,*N*′)palladium(II) 3-methyl­benzoate monohydrate

**DOI:** 10.1107/S1600536810028370

**Published:** 2010-07-21

**Authors:** Mihaela-Diana Şerb, Irmgard Kalf, Ulli Englert

**Affiliations:** a Department of Inorganic Chemistry, Faculty of Applied Chemistry and Materials Science, University Politehnica of Bucharest, Polizu 1, RO-011061 Bucharest, Romania; b Institut für Anorganische Chemie, RWTH Aachen University, Landoltweg 1, 52074 Aachen, Germany

## Abstract

In the title compound, [Pd(C_8_H_10_N)(C_2_H_8_N_2_)](C_8_H_7_O_2_)·H_2_O, the palladium ion is coordinated in a distorted square-planar fashion by the two N atoms from the chelating ethyl­enediamine group and by the N and a C atom of the deprotonated chiral amine. The resulting cationic complex, the 3-methyl­benzoate anion and the hydrate water mol­ecule are inter­connected by N—H⋯O and O—H⋯O hydrogen bonds.

## Related literature

For related organopalladium complexes with chelating oxygen donor ligands, see: Calmuschi & Englert (2002 ▶, 2005*a* ▶,*b* ▶,*c* ▶); Calmuschi *et al.* (2004 ▶). For related organopalladium complexes with nitro­gen donor ligands see: Kalf *et al.* (2006 ▶, 2008 ▶); Şerb *et al.* (2010 ▶). For hydrogen-bond motifs, see: Etter *et al.* (1990 ▶); Etter (1991 ▶).
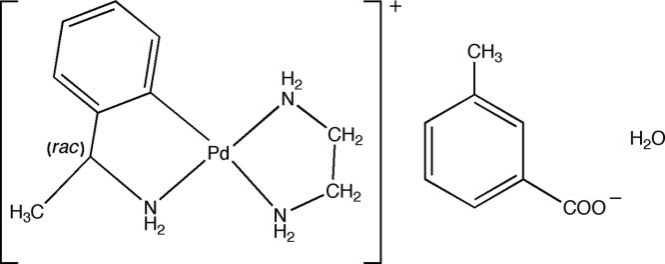


## Experimental

### 

#### Crystal data

[Pd(C_8_H_10_N)(C_2_H_8_N_2_)](C_8_H_7_O_2_)·H_2_O
*M* *_r_* = 439.83
Triclinic, 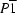

*a* = 7.4787 (4) Å
*b* = 10.7659 (6) Å
*c* = 12.8385 (7) Å
α = 86.1515 (10)°
β = 77.3669 (9)°
γ = 72.5557 (9)°
*V* = 962.28 (9) Å^3^
*Z* = 2
Mo *K*α radiation
μ = 0.99 mm^−1^
*T* = 110 K
0.45 × 0.35 × 0.09 mm

#### Data collection

Bruker SMART CCD area-detector diffractometer
Absorption correction: multi-scan (*MULABS*; Blessing, 1995 ▶; Spek, 2009 ▶) *T*_min_ = 0.666, *T*_max_ = 0.917
10302 measured reflections
4367 independent reflections
4124 reflections with *I* > 2σ(*I*)
*R*_int_ = 0.029

#### Refinement

*R*[*F* ^2^ > 2σ(*F* ^2^)] = 0.023
*wR*(*F* ^2^) = 0.057
*S* = 1.06
4367 reflections
228 parameters
H-atom parameters constrained
Δρ_max_ = 0.61 e Å^−3^
Δρ_min_ = −0.66 e Å^−3^

### 

Data collection: *SMART* (Bruker, 2001 ▶); cell refinement: *SAINT-Plus* (Bruker, 1999 ▶); data reduction: *SAINT-Plus*; program(s) used to solve structure: *SHELXS97* (Sheldrick, 2008 ▶); program(s) used to refine structure: *SHELXL97* (Sheldrick, 2008 ▶); molecular graphics: *PLATON* (Spek, 2009 ▶); software used to prepare material for publication: *SHELXL97*.

## Supplementary Material

Crystal structure: contains datablocks global, I. DOI: 10.1107/S1600536810028370/bt5291sup1.cif

Structure factors: contains datablocks I. DOI: 10.1107/S1600536810028370/bt5291Isup2.hkl

Additional supplementary materials:  crystallographic information; 3D view; checkCIF report

## Figures and Tables

**Table:** 

Pd1—C5	1.9866 (18)
Pd1—N3	2.0325 (15)
Pd1—N2	2.0603 (15)
Pd1—N1	2.1358 (16)

**Table:** 

C5—Pd1—N3	81.77 (7)
C5—Pd1—N2	99.62 (7)
N3—Pd1—N2	175.94 (6)
C5—Pd1—N1	176.54 (6)
N3—Pd1—N1	96.51 (6)
N2—Pd1—N1	82.30 (6)

**Table 2:** Hydrogen-bond geometry (Å, °)

*D*—H⋯*A*	*D*—H	H⋯*A*	*D*⋯*A*	*D*—H⋯*A*
N1—H1*A*⋯O3^i^	0.92	2.17	3.032 (2)	155
N1—H1*B*⋯O1^ii^	0.92	2.25	3.052 (2)	145
N2—H2*B*⋯O3	0.92	2.12	2.958 (2)	151
N3—H3*A*⋯O1^ii^	0.92	2.09	2.947 (2)	153
N3—H3*B*⋯O2	0.92	1.98	2.885 (2)	167
O3—H3*D*⋯O1^iii^	0.84	1.95	2.786 (2)	178
O3—H3*E*⋯O2^i^	0.84	1.89	2.723 (2)	171

Symmetry codes: (i) 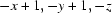; (ii) 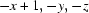; (iii) 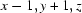.

**Fig. 1.**
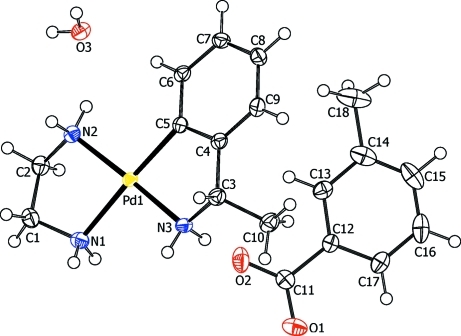
: PLATON (Spek, 2009) plot with displacement ellipsoids at 50% probability; H atoms are represented by spheres of arbitrary radius.

**Fig. 2.**
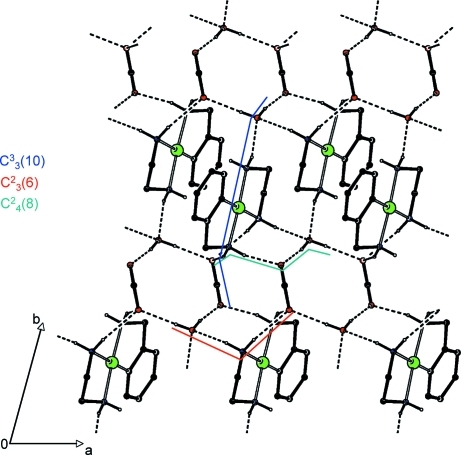
: Hydrogen-bond motifs. The tolyl group of the anion, the methyl group attached to the cation and H atoms attached to carbon have been omitted for clarity.

**Fig. 3.**
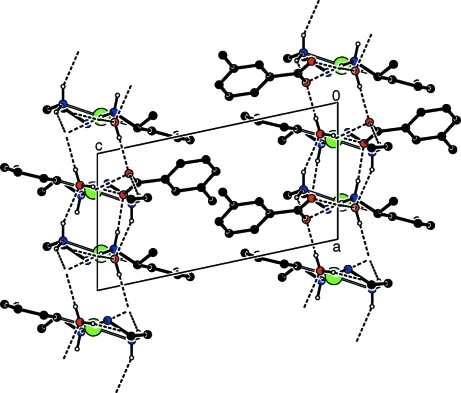
: Packing diagram of the title compound. The dashed lines indicate the hydrogen bonds. H atoms not involved in H bonding have been omitted for clarity.

## supplementary crystallographic information

### Comment

The complex cation (Fig. 1) is essentially square planar: the distance of the metal center to the least-squares plane through the coordinating atoms amounts to 0.00805 (14) Å. The bond lengths between palladium and ethylenediamine nitrogen atoms differ significantly: The Pd—N distance *trans* to carbon is 2.1358 (16) Å and hence longer than the bond to the N donor atom *trans* to the amino group, 2.0603 (15) Å (Table 1). This observation is in agreement with the distance pattern observed for related organopalladium complexes with chelating oxygen donor ligands (Calmuschi & Englert, 2002; Calmuschi *et al.*, 2004; Calmuschi & Englert, 2005*a*; Calmuschi & Englert, 2005*b*; Calmuschi & Englert, 2005*c*) and nitrogen donor ligands (Kalf *et al.*, 2006; Kalf *et al.*, 2008). The title compound forms a two-dimensional network extending in the *a* and *b* directions *via* moderately strong N—H···O and O—H···O hydrogen bonds. With the exception of H2a (attached to N2 of the ethylendiamine ligand) all potential H donors find an acceptor in reasonable geometry for hydrogen bonding (Fig. 2) giving rise to C_4_^2^(8) and C_3_^2^(6) motifs in the *a* direction and C_3_^3^(10) motifs in the *b* direction (Etter *et al.*, 1990; Etter,   1991). The hydrogen bond parameters are presented in Table 2. The flat cationic complexes form stacks extending in [100] direction; the shortest Pd···Pd separation amounts to 4.2157 (3) Å. Figure 3 shows the packing diagram of the title compound. The molecular volume of the title compound (calculated as V/*Z*) is very similar to the molecular volume of {(*rac*)-[2-(1-aminoethyl)phenyl -κ^2^-*C^1^,N*](ethylendiamine)palladium(II)} 3,5-dimethylbenzoate compound reported in our paper (Şerb *et al.*, 2010). The solvent water molecule compensates the smaller size of the anion in the title compound.

### Experimental

46 mg (0.76 mmol) ethylenediamine are added to a solution of 200 mg (0.38 mmol) [{Pd(µ-Cl)(C_6_H_4_CH-MeNH_2_)}_2_] (Calmuschi & Englert, 2002) in 50 ml MeOH at 50 ° C. 185 mg (0.76 mmol) silver-3-methylbenzoate are added; the suspension is stirred for 30 min and allowed to cool to room temperature, and AgCl is removed by filtration. After evaporation of the solvent *in vacuo*, the product is obtained in almost quantitative yield. Slow evaporation of the solvent under ambient conditions gives crystals suitable for X-ray diffraction.

### Refinement

H atoms attached to oxygen were located from difference Fourier map and their bonding distances were idealized to O—H 0.84 Å. They were treated as riding with *U*_iso_(H) = 1.5*U*_eq_(O) and H atoms attached to nitrogen and carbon were calculated and introduced in their idealized positions with C_aryl_—H 0.95 Å, *U*_iso_(H) = 1.2*U*_eq_(C); C_methyl_—H 0.98 Å, *U*_iso_(H) = 1.5*U*_eq_(C); C_ethylene_—H 0.99 Å, *U*_iso_(H) = 1.2*U*_eq_(C) and N—H 0.92 Å, *U*_iso_(H) = 1.2*U*_eq_(N). The methyl groups were allowed to rotate but not to tip. All hydrogen atoms were refined using a riding model.

### Crystal data

**Table:**

[Pd(C_8_H_10_N)(C_2_H_8_N_2_)](C_8_H_7_O_2_)·H_2_O	*Z* = 2
*M**_r_* = 439.83	*F*(000) = 452
Triclinic, *P*1	*D*_x_ = 1.518 Mg m^−^^3^
Hall symbol: -P 1	Mo *K*α radiation, λ = 0.71073 Å
*a* = 7.4787 (4) Å	Cell parameters from 8130 reflections
*b* = 10.7659 (6) Å	θ = 2.6–29.6°
*c* = 12.8385 (7) Å	µ = 0.99 mm^−^^1^
α = 86.1515 (10)°	*T* = 110 K
β = 77.3669 (9)°	Plate, colourless
γ = 72.5557 (9)°	0.45 × 0.35 × 0.09 mm
*V* = 962.28 (9) Å^3^	

### Data collection

**Table:**

Bruker SMART CCD area-detector diffractometer	4367 independent reflections
Radiation source: fine-focus sealed tube	4124 reflections with *I* > 2σ(*I*)
graphite	*R*_int_ = 0.029
ω scans	θ_max_ = 27.5°, θ_min_ = 2.6°
Absorption correction: multi-scan (*MULABS*; Blessing, 1995; Spek, 2009)	*h* = −9→9
*T*_min_ = 0.666, *T*_max_ = 0.917	*k* = −13→12
10302 measured reflections	*l* = −16→16

### Refinement

**Table:**

Refinement on *F*^2^	Primary atom site location: structure-invariant direct methods
Least-squares matrix: full	Secondary atom site location: difference Fourier map
*R*[*F*^2^ > 2σ(*F*^2^)] = 0.023	Hydrogen site location: inferred from neighbouring sites
*wR*(*F*^2^) = 0.057	H-atom parameters constrained
*S* = 1.06	*w* = 1/[σ^2^(*F*_o_^2^) + (0.029*P*)^2^ + 0.250*P*] where *P* = (*F*_o_^2^ + 2*F*_c_^2^)/3
4367 reflections	(Δ/σ)_max_ = 0.001
228 parameters	Δρ_max_ = 0.61 e Å^−^^3^
0 restraints	Δρ_min_ = −0.66 e Å^−^^3^

### Special details

**Table:**

Geometry. All e.s.d.'s (except the e.s.d. in the dihedral angle between two l.s. planes) are estimated using the full covariance matrix. The cell e.s.d.'s are taken into account individually in the estimation of e.s.d.'s in distances, angles and torsion angles; correlations between e.s.d.'s in cell parameters are only used when they are defined by crystal symmetry. An approximate (isotropic) treatment of cell e.s.d.'s is used for estimating e.s.d.'s involving l.s. planes.
Refinement. Refinement of *F*^2^ against ALL reflections. The weighted *R*-factor *wR* and goodness of fit *S* are based on *F*^2^, conventional *R*-factors *R* are based on *F*, with *F* set to zero for negative *F*^2^. The threshold expression of *F*^2^ > σ(*F*^2^) is used only for calculating *R*-factors(gt) *etc*. and is not relevant to the choice of reflections for refinement. *R*-factors based on *F*^2^ are statistically about twice as large as those based on *F*, and *R*- factors based on ALL data will be even larger.

### Fractional atomic coordinates and isotropic or equivalent isotropic displacement parameters (Å^2^)

**Table:**

	*x*	*y*	*z*	*U*_iso_*/*U*_eq_	
Pd1	0.262512 (17)	0.365016 (12)	0.016645 (10)	0.01519 (5)	
N1	0.4171 (2)	0.31370 (15)	−0.14272 (13)	0.0213 (3)	
H1A	0.5441	0.2726	−0.1426	0.026*	
H1B	0.3685	0.2573	−0.1706	0.026*	
N2	0.2346 (2)	0.55102 (15)	−0.04253 (12)	0.0182 (3)	
H2A	0.1152	0.5859	−0.0582	0.022*	
H2B	0.2473	0.6030	0.0078	0.022*	
N3	0.2764 (2)	0.18062 (15)	0.06898 (13)	0.0199 (3)	
H3A	0.2625	0.1339	0.0154	0.024*	
H3B	0.3953	0.1413	0.0840	0.024*	
C1	0.3998 (3)	0.43285 (19)	−0.20908 (15)	0.0236 (4)	
H1C	0.2845	0.4516	−0.2402	0.028*	
H1D	0.5134	0.4199	−0.2683	0.028*	
C2	0.3848 (3)	0.54598 (19)	−0.14036 (16)	0.0228 (4)	
H2C	0.5094	0.5352	−0.1207	0.027*	
H2D	0.3519	0.6284	−0.1807	0.027*	
C3	0.1254 (3)	0.17635 (18)	0.16679 (15)	0.0210 (4)	
H3	0.0072	0.1744	0.1435	0.025*	
C4	0.0812 (2)	0.30251 (18)	0.22643 (15)	0.0186 (4)	
C5	0.1326 (2)	0.40622 (17)	0.16797 (14)	0.0165 (3)	
C6	0.0967 (2)	0.52285 (17)	0.22272 (15)	0.0187 (4)	
H6	0.1316	0.5941	0.1854	0.022*	
C7	0.0114 (3)	0.53561 (18)	0.33022 (15)	0.0219 (4)	
H7	−0.0093	0.6147	0.3660	0.026*	
C8	−0.0441 (3)	0.43363 (19)	0.38601 (15)	0.0230 (4)	
H8	−0.1055	0.4434	0.4593	0.028*	
C9	−0.0090 (3)	0.31723 (18)	0.33368 (15)	0.0218 (4)	
H9	−0.0468	0.2472	0.3714	0.026*	
C10	0.1912 (3)	0.05407 (19)	0.23164 (17)	0.0266 (4)	
H10A	0.2222	−0.0228	0.1869	0.040*	
H10B	0.0885	0.0508	0.2931	0.040*	
H10C	0.3052	0.0554	0.2568	0.040*	
O1	0.8057 (2)	−0.11594 (14)	0.13161 (12)	0.0306 (3)	
O2	0.6595 (2)	0.09451 (16)	0.10904 (12)	0.0341 (4)	
C11	0.7254 (2)	−0.00144 (19)	0.16557 (15)	0.0213 (4)	
C12	0.7075 (2)	0.02334 (18)	0.28219 (15)	0.0188 (4)	
C13	0.6029 (3)	0.14472 (18)	0.32649 (16)	0.0232 (4)	
H13	0.5383	0.2105	0.2832	0.028*	
C14	0.5904 (3)	0.1720 (2)	0.43227 (18)	0.0325 (5)	
C15	0.6858 (3)	0.0748 (3)	0.49358 (18)	0.0377 (5)	
H15	0.6811	0.0921	0.5659	0.045*	
C16	0.7876 (3)	−0.0468 (2)	0.45182 (18)	0.0369 (5)	
H16	0.8507	−0.1125	0.4957	0.044*	
C17	0.7985 (3)	−0.0739 (2)	0.34628 (17)	0.0266 (4)	
H17	0.8675	−0.1581	0.3179	0.032*	
C18	0.4756 (4)	0.3051 (3)	0.4784 (2)	0.0514 (7)	
H18A	0.4177	0.2960	0.5537	0.077*	
H18B	0.3745	0.3451	0.4388	0.077*	
H18C	0.5605	0.3603	0.4727	0.077*	
O3	0.20334 (18)	0.78816 (13)	0.07252 (11)	0.0238 (3)	
H3D	0.0834	0.8155	0.0909	0.036*	
H3E	0.2350	0.8311	0.0183	0.036*	

### Atomic displacement parameters (Å^2^)

**Table:**

	*U*^11^	*U*^22^	*U*^33^	*U*^12^	*U*^13^	*U*^23^
Pd1	0.01435 (7)	0.01388 (8)	0.01678 (8)	−0.00175 (5)	−0.00503 (5)	−0.00128 (5)
N1	0.0200 (7)	0.0219 (8)	0.0217 (8)	−0.0047 (6)	−0.0047 (6)	−0.0035 (6)
N2	0.0156 (7)	0.0191 (8)	0.0209 (8)	−0.0047 (6)	−0.0065 (6)	0.0001 (6)
N3	0.0226 (8)	0.0156 (8)	0.0204 (8)	−0.0019 (6)	−0.0061 (6)	−0.0034 (6)
C1	0.0212 (9)	0.0295 (11)	0.0196 (10)	−0.0064 (8)	−0.0048 (7)	0.0002 (8)
C2	0.0210 (9)	0.0247 (10)	0.0230 (10)	−0.0082 (7)	−0.0043 (7)	0.0030 (8)
C3	0.0230 (9)	0.0182 (9)	0.0232 (10)	−0.0068 (7)	−0.0070 (7)	0.0001 (7)
C4	0.0172 (8)	0.0178 (9)	0.0217 (10)	−0.0032 (7)	−0.0086 (7)	−0.0005 (7)
C5	0.0137 (7)	0.0183 (9)	0.0177 (9)	−0.0026 (6)	−0.0066 (6)	−0.0004 (7)
C6	0.0178 (8)	0.0167 (9)	0.0222 (9)	−0.0038 (7)	−0.0074 (7)	0.0004 (7)
C7	0.0237 (9)	0.0193 (9)	0.0223 (10)	−0.0023 (7)	−0.0083 (7)	−0.0048 (7)
C8	0.0236 (9)	0.0260 (10)	0.0176 (9)	−0.0031 (8)	−0.0059 (7)	−0.0015 (7)
C9	0.0239 (9)	0.0212 (10)	0.0214 (10)	−0.0068 (7)	−0.0080 (7)	0.0040 (7)
C10	0.0302 (10)	0.0197 (10)	0.0314 (11)	−0.0086 (8)	−0.0084 (8)	0.0025 (8)
O1	0.0268 (7)	0.0314 (8)	0.0339 (8)	−0.0081 (6)	−0.0037 (6)	−0.0131 (6)
O2	0.0269 (7)	0.0463 (10)	0.0246 (8)	−0.0042 (7)	−0.0081 (6)	0.0102 (7)
C11	0.0136 (8)	0.0284 (10)	0.0227 (10)	−0.0076 (7)	−0.0032 (7)	−0.0013 (8)
C12	0.0177 (8)	0.0192 (9)	0.0216 (10)	−0.0080 (7)	−0.0055 (7)	0.0030 (7)
C13	0.0218 (9)	0.0196 (10)	0.0276 (11)	−0.0083 (7)	−0.0013 (7)	0.0017 (8)
C14	0.0300 (11)	0.0368 (12)	0.0323 (12)	−0.0178 (9)	0.0044 (9)	−0.0114 (9)
C15	0.0389 (12)	0.0616 (16)	0.0210 (11)	−0.0279 (12)	−0.0040 (9)	−0.0046 (10)
C16	0.0345 (11)	0.0505 (15)	0.0305 (12)	−0.0166 (11)	−0.0158 (9)	0.0173 (10)
C17	0.0228 (9)	0.0242 (10)	0.0328 (11)	−0.0061 (8)	−0.0087 (8)	0.0061 (8)
C18	0.0485 (15)	0.0466 (16)	0.0556 (17)	−0.0184 (12)	0.0105 (12)	−0.0275 (13)
O3	0.0220 (6)	0.0194 (7)	0.0277 (8)	−0.0038 (5)	−0.0036 (5)	0.0009 (5)

### Geometric parameters (Å, °)

**Table:**

Pd1—C5	1.9866 (18)	C7—H7	0.9500
Pd1—N3	2.0325 (15)	C8—C9	1.389 (3)
Pd1—N2	2.0603 (15)	C8—H8	0.9500
Pd1—N1	2.1358 (16)	C9—H9	0.9500
N1—C1	1.480 (2)	C10—H10A	0.9800
N1—H1A	0.9200	C10—H10B	0.9800
N1—H1B	0.9200	C10—H10C	0.9800
N2—C2	1.483 (2)	O1—C11	1.259 (2)
N2—H2A	0.9200	O2—C11	1.260 (2)
N2—H2B	0.9200	C11—C12	1.508 (3)
N3—C3	1.502 (2)	C12—C13	1.391 (3)
N3—H3A	0.9200	C12—C17	1.392 (3)
N3—H3B	0.9200	C13—C14	1.386 (3)
C1—C2	1.514 (3)	C13—H13	0.9500
C1—H1C	0.9900	C14—C15	1.383 (3)
C1—H1D	0.9900	C14—C18	1.515 (3)
C2—H2C	0.9900	C15—C16	1.378 (3)
C2—H2D	0.9900	C15—H15	0.9500
C3—C4	1.517 (3)	C16—C17	1.385 (3)
C3—C10	1.521 (3)	C16—H16	0.9500
C3—H3	1.0000	C17—H17	0.9500
C4—C9	1.391 (3)	C18—H18A	0.9800
C4—C5	1.406 (3)	C18—H18B	0.9800
C5—C6	1.406 (3)	C18—H18C	0.9800
C6—C7	1.385 (3)	O3—H3D	0.8401
C6—H6	0.9500	O3—H3E	0.8400
C7—C8	1.389 (3)		
			
C5—Pd1—N3	81.77 (7)	C4—C5—Pd1	114.64 (13)
C5—Pd1—N2	99.62 (7)	C7—C6—C5	121.12 (17)
N3—Pd1—N2	175.94 (6)	C7—C6—H6	119.4
C5—Pd1—N1	176.54 (6)	C5—C6—H6	119.4
N3—Pd1—N1	96.51 (6)	C6—C7—C8	120.47 (18)
N2—Pd1—N1	82.30 (6)	C6—C7—H7	119.8
C1—N1—Pd1	109.38 (11)	C8—C7—H7	119.8
C1—N1—H1A	109.8	C7—C8—C9	119.39 (18)
Pd1—N1—H1A	109.8	C7—C8—H8	120.3
C1—N1—H1B	109.8	C9—C8—H8	120.3
Pd1—N1—H1B	109.8	C8—C9—C4	120.47 (18)
H1A—N1—H1B	108.2	C8—C9—H9	119.8
C2—N2—Pd1	108.98 (11)	C4—C9—H9	119.8
C2—N2—H2A	109.9	C3—C10—H10A	109.5
Pd1—N2—H2A	109.9	C3—C10—H10B	109.5
C2—N2—H2B	109.9	H10A—C10—H10B	109.5
Pd1—N2—H2B	109.9	C3—C10—H10C	109.5
H2A—N2—H2B	108.3	H10A—C10—H10C	109.5
C3—N3—Pd1	112.76 (11)	H10B—C10—H10C	109.5
C3—N3—H3A	109.0	O1—C11—O2	124.72 (19)
Pd1—N3—H3A	109.0	O1—C11—C12	117.85 (17)
C3—N3—H3B	109.0	O2—C11—C12	117.42 (17)
Pd1—N3—H3B	109.0	C13—C12—C17	119.18 (18)
H3A—N3—H3B	107.8	C13—C12—C11	120.29 (17)
N1—C1—C2	109.21 (16)	C17—C12—C11	120.52 (17)
N1—C1—H1C	109.8	C14—C13—C12	121.58 (19)
C2—C1—H1C	109.8	C14—C13—H13	119.2
N1—C1—H1D	109.8	C12—C13—H13	119.2
C2—C1—H1D	109.8	C15—C14—C13	118.1 (2)
H1C—C1—H1D	108.3	C15—C14—C18	121.4 (2)
N2—C2—C1	109.42 (15)	C13—C14—C18	120.5 (2)
N2—C2—H2C	109.8	C16—C15—C14	121.3 (2)
C1—C2—H2C	109.8	C16—C15—H15	119.4
N2—C2—H2D	109.8	C14—C15—H15	119.4
C1—C2—H2D	109.8	C15—C16—C17	120.4 (2)
H2C—C2—H2D	108.2	C15—C16—H16	119.8
N3—C3—C4	106.57 (15)	C17—C16—H16	119.8
N3—C3—C10	110.65 (15)	C16—C17—C12	119.4 (2)
C4—C3—C10	114.39 (16)	C16—C17—H17	120.3
N3—C3—H3	108.4	C12—C17—H17	120.3
C4—C3—H3	108.4	C14—C18—H18A	109.5
C10—C3—H3	108.4	C14—C18—H18B	109.5
C9—C4—C5	120.81 (17)	H18A—C18—H18B	109.5
C9—C4—C3	122.18 (17)	C14—C18—H18C	109.5
C5—C4—C3	117.00 (16)	H18A—C18—H18C	109.5
C6—C5—C4	117.69 (17)	H18B—C18—H18C	109.5
C6—C5—Pd1	127.58 (14)	H3D—O3—H3E	106.6
			
C5—Pd1—N1—C1	−130.9 (10)	N3—Pd1—C5—C4	−11.99 (12)
N3—Pd1—N1—C1	169.22 (12)	N2—Pd1—C5—C4	164.14 (12)
N2—Pd1—N1—C1	−6.87 (12)	N1—Pd1—C5—C4	−72.3 (11)
C5—Pd1—N2—C2	157.14 (12)	C4—C5—C6—C7	−0.8 (2)
N3—Pd1—N2—C2	−93.1 (8)	Pd1—C5—C6—C7	−177.17 (13)
N1—Pd1—N2—C2	−19.95 (12)	C5—C6—C7—C8	−1.2 (3)
C5—Pd1—N3—C3	23.45 (12)	C6—C7—C8—C9	1.6 (3)
N2—Pd1—N3—C3	−86.9 (8)	C7—C8—C9—C4	0.1 (3)
N1—Pd1—N3—C3	−159.57 (12)	C5—C4—C9—C8	−2.2 (3)
Pd1—N1—C1—C2	31.87 (17)	C3—C4—C9—C8	178.88 (17)
Pd1—N2—C2—C1	43.43 (17)	O1—C11—C12—C13	−173.31 (17)
N1—C1—C2—N2	−50.20 (19)	O2—C11—C12—C13	7.0 (3)
Pd1—N3—C3—C4	−28.71 (17)	O1—C11—C12—C17	8.0 (3)
Pd1—N3—C3—C10	−153.65 (13)	O2—C11—C12—C17	−171.65 (17)
N3—C3—C4—C9	−161.25 (16)	C17—C12—C13—C14	1.4 (3)
C10—C3—C4—C9	−38.6 (2)	C11—C12—C13—C14	−177.30 (17)
N3—C3—C4—C5	19.8 (2)	C12—C13—C14—C15	0.1 (3)
C10—C3—C4—C5	142.38 (17)	C12—C13—C14—C18	179.93 (19)
C9—C4—C5—C6	2.5 (2)	C13—C14—C15—C16	−1.2 (3)
C3—C4—C5—C6	−178.52 (15)	C18—C14—C15—C16	179.0 (2)
C9—C4—C5—Pd1	179.33 (13)	C14—C15—C16—C17	0.8 (3)
C3—C4—C5—Pd1	−1.7 (2)	C15—C16—C17—C12	0.7 (3)
N3—Pd1—C5—C6	164.48 (16)	C13—C12—C17—C16	−1.8 (3)
N2—Pd1—C5—C6	−19.39 (16)	C11—C12—C17—C16	176.90 (17)
N1—Pd1—C5—C6	104.2 (10)		

### Hydrogen-bond geometry (Å, °)

**Table:**

*D*—H···*A*	*D*—H	H···*A*	*D*···*A*	*D*—H···*A*
N1—H1A···O3^i^	0.92	2.17	3.032 (2)	155
N1—H1B···O1^ii^	0.92	2.25	3.052 (2)	145
N2—H2B···O3	0.92	2.12	2.958 (2)	151
N3—H3A···O1^ii^	0.92	2.09	2.947 (2)	153
N3—H3B···O2	0.92	1.98	2.885 (2)	167
O3—H3D···O1^iii^	0.84	1.95	2.786 (2)	178
O3—H3E···O2^i^	0.84	1.89	2.723 (2)	171

Symmetry codes: (i) −*x*+1, −*y*+1, −*z*; (ii) −*x*+1, −*y*, −*z*; (iii) *x*−1, *y*+1, *z*.
